# A Multimodal UAV-IoT Sensing Framework for Intelligent Pest Density Estimation in Smart Agricultural Systems

**DOI:** 10.3390/s26092877

**Published:** 2026-05-05

**Authors:** Yida Zhang, Jianxi Chen, Xin Zeng, Runxi Chen, Lirui Chen, Shanhe Xiao, Yihong Song

**Affiliations:** 1 China Agricultural University, Beijing 100083, China; 2 Central University of Finance and Economics, Beijing 100081, China; 3 Peking University, Beijing 100871, China

**Keywords:** AI-driven sensing, multimodal sensor fusion, UAV–IoT integration, intelligent environmental perception, smart sensing agriculture systems

## Abstract

Accurate estimation of dynamic environmental phenomena through intelligent sensing systems plays a critical role in enabling reliable monitoring and decision-making in complex real-world scenarios. With the rapid development of artificial intelligence-driven sensing technologies and Internet of Things systems, modern agricultural monitoring is evolving from isolated data acquisition toward intelligent, multimodal perception and decision-making. However, traditional approaches predominantly rely on single data sources, making it difficult to simultaneously capture plant phenotypic variations and environment-driven mechanisms, thereby limiting model applicability in complex field scenarios. To address this issue, a multimodal pest density estimation framework, namely the Pest Density Estimation Framework (PDEF), is proposed, which integrates UAV-based imagery, trap monitoring data, and environmental sensor measurements. In this framework, crop canopy damage features are extracted using convolutional neural networks, while temporal encoding is employed to model dynamic environmental variations. Cross-modal feature alignment and environment-aware enhancement mechanisms are further introduced to achieve deep integration of multi-source information, enabling the construction of a unified feature representation space and improving estimation accuracy. Extensive experiments conducted on a constructed multimodal agricultural dataset demonstrate that the proposed method achieves MAE, RMSE, and MAPE values of 5.47, 7.62, and 14.9%, respectively, significantly outperforming the Transformer-based fusion model (MAE 6.01, RMSE 8.16). Meanwhile, the coefficient of determination reaches $R^{2}=0.84$, indicating superior fitting capability and stability. In multimodal combination experiments, the three-modality fusion reduces error metrics by more than 20% on average compared with single-modality models, validating the effectiveness of multi-source collaborative modeling. From the perspective of integrating plant phenotypic analysis and environmental perception, this study provides a novel AI-driven intelligent sensing framework for pest monitoring and crop management, contributing to improved pest prediction capability and enhanced intelligence in agricultural production systems. This study further provides practical implications for agricultural economics and supply chain optimization by enabling data-driven decision-making through intelligent sensing systems.

## 1. Introduction

In modern agricultural production systems, the achievement of high yield and superior quality in crops is highly dependent on precise monitoring of pests and diseases [1]. Among these, pest monitoring serves as a core component of integrated pest management (IPM) [2], guiding control decisions and enhancing sustainability [3]. While traditional manual surveys and trap-based counting provide local accuracy, they suffer from high labor intensity and limited spatial coverage [4]. Recent advancements in unmanned aerial vehicle (UAV) remote sensing have enabled efficient field-scale imagery acquisition [5,6], providing detailed observations of crop canopy damage and pest-affected regions through visual cues [7,8].

However, significant limitations remain in existing studies. We systematically identify three fundamental defects in current methodologies. First, regarding task objectives, most deep learning approaches focus on object-level detection or species classification [9,10,11,12], which cannot directly support agricultural decision-making that requires field-scale population density D to determine control thresholds [13,14]. Second, from the perspective of data modalities, existing methods predominantly rely on single visual sources and overlook the synergistic effects of environmental driving factors, such as temperature and humidity, which are typically collected via IoT sensors [15,16,17]. Third, in terms of feature representation, current frameworks lack the capability to jointly model spatial imagery and temporal time series, leading to semantic discrepancies and scale inconsistencies that hinder effective multi-source fusion [18].

To address the aforementioned challenges, a pest density estimation framework, termed the Pest Density Estimation Framework (PDEF), is proposed by integrating UAV imagery, trap-based monitoring data, and environmental sensor measurements. The framework establishes a unified multimodal data-driven modeling paradigm with density estimation as the primary objective. Specifically, crop damage features are extracted from UAV imagery, while trap-based counts provide localized references, and environmental sensors capture ecological driving factors. To effectively address inconsistencies in scale and semantics, a cross-modal feature alignment mechanism and an environment-aware enhancement strategy are designed, thereby improving information synergy and model adaptability.

The main contributions of this study are summarized as follows:

1. A systematic multimodal modeling paradigm for pest density estimation is established, which integrates the construction of a multi-source agricultural dataset with the proposal of the Pest Density Estimation Framework (PDEF) to provide a unified data-driven foundation for field-scale population monitoring.
2. Innovative architectural components, including a cross-modal feature alignment module and an environment-aware enhancement module, are designed to effectively mitigate discrepancies across heterogeneous data sources in spatial, temporal, and semantic dimensions.
3. Extensive experimental evaluations are conducted under real-world monitoring conditions to demonstrate that the proposed PDEF framework significantly outperforms traditional object-level detection approaches and single-modality prediction models in terms of accuracy and robustness.
4. The practical application value of the proposed framework is further illustrated by its capability to provide data-driven insights for precision resource allocation and risk-aware decision-making in intelligent agricultural management.

In summary, this research establishes a novel multimodal paradigm for intelligent pest density estimation that is fundamentally differentiated from conventional vision-only detection methods. By synergizing UAV-based spatial imagery with trap-based localized counts and IoT-driven environmental dynamics within the proposed PDEF framework, we enable precise field-scale population monitoring. The core innovations lie in the design of cross-modal feature alignment and environment-aware enhancement mechanisms, which effectively overcome the data heterogeneity and semantic gaps that have previously limited multi-source agricultural sensing. This integrated approach provides a robust technical pathway for data-driven decision support in precision agriculture.

## 2. Related Work

### 2.1. Vision-Based Agricultural Pest Detection Methods

In recent years, with the rapid development of deep learning techniques [19], vision-based agricultural pest detection methods have achieved significant progress. A large body of research has employed convolutional neural networks (CNN) and their variants to automatically identify and localize pests in trap images or crop leaf images [20]. For example, methods based on two-stage detection frameworks, such as Faster R-CNN, are capable of achieving high-precision pest detection [21,22], while single-stage detectors represented by the YOLO series provide a favorable trade-off between real-time performance and detection accuracy [23,24]. In addition, some studies have incorporated fine-grained classification networks to improve discrimination among different pest species [25]. Despite their strong performance in pest recognition and counting tasks, these approaches primarily focus on classification or object-level counting, which remains misaligned with the core requirements of agricultural production [26]. In practical scenarios, greater attention is paid to the severity of pest infestation at the field scale, namely the distribution of pest population density, rather than detection results from individual images [27]. Existing methods are typically based on local image observations and lack the capability to model spatial-scale extension, making it difficult to reflect the overall pest dynamics across farmland [28]. Furthermore, current vision-based methods generally exhibit several limitations. First, they rely solely on visual information while neglecting the ecological context of pest occurrence. Second, detection or counting results cannot be directly mapped to actual pest population density, denoted as *D*. Third, key factors such as meteorological conditions and micro-environmental variations are not explicitly modeled, which limits the generalization ability of these methods in complex agricultural scenarios. Consequently, purely vision-driven pest detection approaches remain insufficient for supporting precision agricultural decision-making.

### 2.2. Remote Sensing and UAV-Based Agricultural Monitoring

The emergence of unmanned aerial vehicle (UAV) remote sensing technology has provided high spatiotemporal resolution data acquisition capabilities for agricultural monitoring [29]. Compared with traditional satellite remote sensing, UAV platforms can acquire centimeter-level resolution imagery at relatively low cost [30] and have been widely applied in tasks such as crop growth assessment, disease detection, nitrogen status monitoring, and field phenotyping [31]. In these studies, vegetation indices, texture features, or deep features are typically extracted to model crop health conditions, enabling fine-grained characterization of spatial heterogeneity in farmland [32]. In the context of pest monitoring, several studies have begun to explore the use of UAV imagery for identifying crop damage regions or stress conditions [33]. For instance, pest occurrence can be indirectly inferred by detecting changes in leaf color, canopy sparsity, or abnormal texture distributions [34]. Such approaches enable the localization of potentially affected areas at large scales, providing useful guidance for field inspection and pest control [35]. However, the application of UAV remote sensing in pest monitoring still faces several critical challenges. First, UAV imagery primarily reflects visual changes in crop appearance, and its representation of pest infestation is inherently indirect, making it difficult to distinguish between different types of stress, such as disease, drought, or nutrient deficiency [36]. Second, remote sensing-based methods are typically designed for region-level classification or segmentation tasks and lack the capability for quantitative modeling of pest population scale. Therefore, relying solely on UAV imagery is insufficient for accurate estimation of pest population density.

### 2.3. Multimodal Agricultural Sensor Data Fusion

With the development of the Internet of Things (IoT) in agriculture [37], multi-source sensing data have been increasingly utilized in agricultural production. By deploying weather stations, soil sensors, and crop monitoring devices in farmland, environmental variables such as temperature, humidity, light intensity, wind speed, and soil moisture can be continuously collected [38]. These data provide essential support for crop growth modeling and agricultural prediction tasks [39]. In recent years, efforts have been made to integrate multimodal data, including imagery, meteorological information, and time-series sensor data, to improve the performance of agricultural applications such as yield prediction, irrigation management, and disease warning [40]. At the methodological level, multimodal fusion is commonly achieved through feature-level fusion, decision-level fusion, or joint representation learning based on deep learning models [41]. For example, convolutional neural networks are combined with recurrent neural networks (RNN) or Transformer architectures to jointly model spatial and temporal information, thereby enhancing predictive performance [42]. These studies demonstrate that multi-source data fusion can effectively compensate for the limitations of single data sources and improve model robustness. Nevertheless, multimodal fusion research in agricultural pest monitoring remains relatively limited. Existing studies are predominantly focused on single modalities, such as imagery or meteorological data [43], and lack systematic investigation of cross-source interactions. In particular, for pest population density estimation tasks, a unified deep learning-based modeling framework is still lacking. Moreover, significant discrepancies exist among UAV imagery, trap-based counting data, and environmental sensor measurements in terms of spatial scale, temporal resolution, and data distribution [44], making effective alignment and fusion a challenging yet unresolved problem.

### 2.4. Data-Driven Agri-Economics

In recent years, the integration of agricultural economics and agricultural supply chain management with intelligent sensing and data-driven technologies has attracted increasing attention [45,46]. Traditional agricultural supply chains often suffer from information asymmetry, delayed feedback, and limited end-to-end visibility across production, distribution, and consumption stages, which lead to inefficient resource allocation and increased operational risks [47]. With the advancement of IoT-based sensing systems and artificial intelligence-driven analytics, real-time data collected from field environments, logistics processes, and market dynamics can be effectively integrated to support decision-making in agricultural production and circulation [48]. Existing studies have explored the use of sensor networks, remote sensing technologies, and multi-source data fusion methods to improve demand forecasting, inventory management, and risk assessment in agricultural supply chains [49]. However, most existing approaches still rely on coarse-grained data or single-modality information, lacking a fine-grained perception of field-scale dynamics and their interactions with environmental factors. Therefore, developing intelligent sensing frameworks that bridge field-level monitoring with supply chain-level decision-making has become a critical research direction for enhancing the efficiency and resilience of modern agricultural systems.

## 3. Materials and Method

### 3.1. Data Collection

In this study, to achieve accurate modeling of pest population density at the field scale, a multimodal agricultural pest monitoring dataset integrating multi-source information was constructed, as shown in Table 1. The data collection region is located in Bayannur City, Inner Mongolia Autonomous Region, and partial supplementary data were collected from online sources. This region has relatively stable crop distributions and representative pest occurrence patterns. The overall data collection period covered key stages of the crop growing season, spanning from April 2023 to October 2024, including early, middle, and peak stages of pest outbreaks, thereby ensuring temporal completeness and representativeness of the dataset. During the data acquisition process, UAV remote sensing, field-based trap monitoring, and environmental sensor measurements were jointly utilized to form a multimodal observation system, enabling comprehensive characterization of the spatial distribution of pest occurrence and its environmental driving mechanisms.

**Figure 1:**
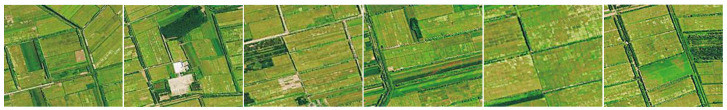
The illuminations of the UAV RGB image dataset.

UAV imagery data were acquired using a multi-rotor UAV platform equipped with a high-resolution RGB camera, which was deployed for periodic aerial surveys over farmland regions, as shown in Figure 1. Flight missions were executed along predefined routes, with flight altitude controlled within the range of 20–40 m, corresponding to a ground sampling resolution of approximately 1–3 cm. Image acquisition was primarily conducted under clear and low-wind conditions between 10:00 and 15:00 to minimize the influence of illumination variation and shadow effects. To ensure spatial continuity and sufficient coverage, adjacent flight paths were designed with approximately 70% forward overlap and 60% side overlap. The collected raw images were further processed through mosaicking and cropping operations to generate standardized field image patches for subsequent visual feature extraction and spatial modeling. These imagery data primarily captured crop canopy structure, leaf color variation, and damaged patch distribution, serving as a key data source for representing the spatial heterogeneity of pest infestation.

Trap-based insect counting data were obtained through the deployment of pheromone traps across the farmland. The traps were arranged in a grid-like pattern within the study area, with spacing between adjacent units maintained at approximately 30–50 m to ensure effective sampling of pest distribution. Each trap was assigned a unique identifier and was manually inspected at fixed intervals, typically every 3–5 days, during which captured insects were counted. To derive the ground truth pest density D, we established a mapping relationship where discrete trap counts are normalized by the effective pheromone attraction area to calculate the number of individuals per square meter. To verify this trap-derived density, we conducted synchronized manual field surveys using a standard five-point sampling method on crop leaves within a 5-m radius of each trap. A high Pearson correlation coefficient (r > 0.88) was observed between the manual survey results and the trap records, validating the accuracy of using trap data as a representative reference for field-scale density. The recorded counts represent the number of target pests captured per unit time and are used as an important observational reference for pest population density. To mitigate the influence of stochastic fluctuations, temporal smoothing was applied to the raw counting sequences. Furthermore, by incorporating the spatial locations of traps, point-based observations were mapped to corresponding farmland regions, thereby strengthening spatial correspondence with UAV imagery.

Regarding data annotation and quality control, a team of six agricultural experts and trained graduate students from agricultural universities performed the labeling of crop damage features and pest density levels. We established standardized annotation rules where damage severity was categorized into four levels based on the ratio of damaged leaf area and canopy sparsity observed in UAV patches. To ensure high-quality annotations, a three-stage quality control procedure was implemented: initially, each data sample was independently annotated by two individuals; subsequently, a cross-validation check was performed, and any samples with conflicting labels were reviewed by a senior expert to reach a final consensus. This rigorous process achieved an inter-annotator agreement rate of over 92%, ensuring the reliability of the labels used for model training.

To provide a quantitative characterization of the dataset, we analyzed the statistical distribution of the 12,000 trap records across different pest density intervals. The samples were categorized into three levels: low density (0–20 individuals per trap), medium density (21–50 individuals per trap), and high density (exceeding 50 individuals per trap), which accounted for 42.5%, 35.8%, and 21.7% of the total data, respectively. This distribution ensured that the PDEF framework was trained on diverse infestation scenarios. Regarding data completeness, the synchronization rate across UAV imagery, IoT sensors, and trap counts reached 98.2%, with minor data gaps primarily caused by extreme weather conditions during the two-year collection period. The spatial coverage encompassed over 15 representative field plots in Bayannur, covering a total experimental area of approximately 120 hectares, which provides the necessary spatial heterogeneity for robust model training and validation.

Environmental sensor data were collected from field-deployed microweather stations and distributed sensor nodes. The sensor types included temperature, relative humidity, wind speed, rainfall, and illumination intensity, with sampling intervals typically ranging from 10 to 30 min, resulting in continuous time-series data. All sensing devices were calibrated prior to deployment to ensure measurement accuracy and consistency. During data acquisition, measurements were transmitted in real time to a centralized data platform via wireless communication modules for unified storage and management. These environmental data were used to characterize the ecological driving factors of pest occurrence and propagation, providing essential temporal dynamic information for the modeling process.

### 3.2. Data Preprocessing and Augmentation Strategy

In multimodal agricultural pest monitoring tasks, significant discrepancies exist among different data sources in terms of sampling strategies, spatial resolution, and temporal scales. Such heterogeneity directly affects training stability and prediction performance of the model. Therefore, prior to constructing a unified pest density estimation framework, systematic preprocessing and data augmentation are required for UAV imagery, environmental sensor time-series data, and trap-based pest count data. The objective of data preprocessing is to map raw observations into a feature space more suitable for model learning through a series of mathematical transformations, while data augmentation aims to approximate real-world data variability by introducing controlled perturbations, thereby enhancing model robustness in complex agricultural scenarios. For UAV imagery data, preprocessing primarily focuses on spatial normalization and pixel distribution standardization. Since raw aerial images typically possess high resolution and contain substantial redundant background information, a sliding window or region cropping strategy is first applied to partition images into fixed-size patches. Let the original image be denoted as $X\in R^{H\times W\times C}$, where *H* and *W* represent image height and width, and *C* denotes the number of channels. The cropping operation can be formulated as

(1) $$X_{i}=C\left(X;x_{i},y_{i},h,w\right),$$

where C(·) denotes the cropping function, $\left(x_{i},y_{i}\right)$ represents the top-left coordinate of the cropping window, and *h* and *w* denote the height and width of the cropped region. This operation effectively reduces background interference and enhances the representation of pest-related features in local regions. Subsequently, to mitigate variations caused by illumination conditions and sensor responses, pixel values are normalized using a standardization approach based on mean and standard deviation:

(2) $$\hat{X}=\frac{X-\mu }{\sigma },$$

where μ and σ denote the global mean and standard deviation of the dataset, respectively. This transformation facilitates faster convergence and improves numerical stability. To further enhance adaptability to complex agricultural environments, multiple data augmentation strategies are introduced to simulate spatial and illumination variations. For geometric transformations, rotation is applied to simulate changes in UAV flight orientation, which can be expressed as

(3) $$\left[\begin{array}{c} x^{\prime }\\ y^{\prime } \end{array}\right]=\left[\begin{array}{cc} \cos\theta & -\sin\theta \\ \sin\theta & \cos\theta \end{array}\right]\left[\begin{array}{c} x\\ y \end{array}\right],$$

where θ denotes the rotation angle. Flipping operations, including horizontal and vertical mirroring, are employed to increase spatial diversity. For brightness adjustment, a linear transformation is adopted:

(4) $$X^{\prime }=\alpha X+\beta ,$$

where α controls contrast, and β represents brightness offset. These augmentation strategies effectively construct a local perturbation space around the original data distribution, thereby improving model generalization to unseen scenarios. For environmental sensor time-series data, preprocessing focuses on temporal alignment and missing value handling. Due to differences in sampling frequencies across sensors, time interpolation is applied to align multi-source data onto a unified temporal scale. Let the original time series be denoted as ${\left\{E\left(t_{k}\right)\right\}}_{k=1}^{N}$, where $E(t_{k})$ represents the observed value of an environmental variable at the discrete time point $t_{k}$. For an arbitrary target time point *t* satisfying $t_{i}\leq t\leq t_{i+1}$, the corresponding estimated value E(t) is calculated using linear interpolation:

(5) $$E\left(t\right)=E\left(t_{i}\right)+\frac{t-t_{i}}{t_{i+1}-t_{i}}\left(E\left(t_{i+1}\right)-E\left(t_{i}\right)\right),$$

where $t_{i}$ and $t_{i+1}$ denote the two consecutive recorded timestamps immediately preceding and following the target time *t*, and $E\left(t_{i}\right),E\left(t_{i+1}\right)$ are the corresponding sensor measurements at those instances. This method ensures temporal continuity while reducing inconsistencies caused by irregular sampling.

To address missing values commonly present in sensor data, neighborhood-based imputation strategies are adopted. Specifically, for a missing observation at time $t_{j}$, the value $E(t_{j})$ is estimated by calculating the arithmetic mean of valid observations within a predefined temporal neighborhood $\Omega_{j}$:

(6) $$E\left(t_{j}\right)=\frac{1}{K}\sum_{t_{k}\in \Omega_{j}}E\left(t_{k}\right),$$

where $\Omega_{j}=\left\{t_{k}|\left|t_{j}-t_{k}\right|\leq \delta ,k\neq j\right\}$ represents the set of *K* available neighboring data points within a temporal window δ. Here, *K* denotes the cardinality of the set $\Omega_{j}$, and $E(t_{k})$ represents the valid samples used for imputation. Furthermore, to eliminate scale differences among environmental variables, standardization is performed:

(7) $$\hat{E}=\frac{E-\mu_{E}}{\sigma_{E}},$$

where $\mu_{E}$ and $\sigma_{E}$ denote the mean and standard deviation of the corresponding variable. This transformation enables different environmental variables to contribute to model training under a unified scale, preventing any single variable from dominating the learning process. For trap-based pest count data, which are inherently discrete observations and susceptible to stochastic noise and sampling errors, smoothing is applied to extract underlying trends. A commonly used approach is moving average filtering, formulated as

(8) $${\tilde{C}}_{t}=\frac{1}{2k+1}\sum_{i=-k}^{k}C_{t+i},$$

where ${\tilde{C}}_{t}$ denotes the smoothed pest count at time *t*, and *k* represents the window radius. This method effectively suppresses high-frequency noise and ensures that the data better reflect the continuous dynamics of pest populations. From a probabilistic modeling perspective, data preprocessing and augmentation can be interpreted as a reconstruction process of the original data distribution p(x). By introducing a set of transformation functions T, the augmented data distribution can be expressed as

(9) $$p_{aug}\left(x\right)=\int p\left(x\right),p\left(T\right),dT.$$

This formulation indicates that data augmentation effectively constructs a smoother and more generalized distribution around the original data, enabling the model to learn more robust feature representations.

### 3.3. Proposed Method

#### 3.3.1. Overall

After multimodal data preprocessing and alignment are completed, the processed data are uniformly fed into the proposed Pest Density Estimation Framework (PDEF) for modeling. The pipeline implements a hierarchical fusion strategy that transforms raw multi-source observations into high-dimensional feature representations. Specifically, visual spatial patterns are captured by a convolutional backbone, while environmental dynamics and local counts are encoded into a shared embedding space. These heterogeneous features undergo cross-modal alignment and environment-aware modulation to ensure semantic consistency before being mapped to the final density prediction. The entire process is shown in Algorithm 1.
**Algorithm 1** Inference Pathway of PDEF1:**Input:** UAV image *X*, IoT sequence *E*, trap counts *C*2:**Output:** Estimated pest density *y*3:$F_{v}\leftarrow \mathrm{ConvEncoder}\left(X\right)$4:$F_{e}\leftarrow \mathrm{TempEncoder}\left(E\right)$5:$F_{t}\leftarrow \mathrm{MapEmbedding}\left(C\right)$6:$Z_{\mathrm{align}}\leftarrow \mathrm{CrossModalAttn}\left(Q=F_{v},\;K=\left[F_{e};F_{t}\right],\;V=\left[F_{e};F_{t}\right]\right)$7:$\gamma ,\beta \leftarrow \mathrm{GenerateModulation}(F_{e})$8:$F_{v,\mathrm{enhanced}}\leftarrow F_{v}⊙\left(1+\gamma \right)+\beta$9:$g\leftarrow \mathrm{GlobalAggregate}(F_{v,\mathrm{enhanced}},Z_{\mathrm{align}})$10:y←MLPRegression(g)11:**return** *y*

#### 3.3.2. Cross-Modal Feature Alignment Module

In the proposed PDEF framework, the core objective of the cross-modal feature alignment module is to alleviate inconsistencies in semantic space and scale representation among UAV visual features, environmental sensor features, and trap-based count features and to construct a unified feature interaction and alignment pathway based on a multi-head cross-modal attention mechanism.

**Figure 2:**
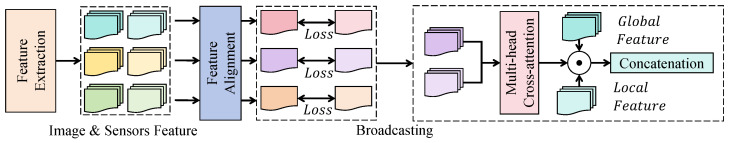
The cross-modal feature alignment module performs semantic alignment and information interaction among UAV visual features, environmental temporal features, and trap-count features through multi-head cross-attention and feature broadcasting.

As shown in Figure 2, the feature representation from the visual encoder is first denoted as $F_{uav}\in R^{N\times d}$, the environmental temporal encoding feature is denoted as $F_{env}\in R^{T\times d}$, and the trap embedding feature is denoted as $F_{trap}\in R^{M\times d}$, where *d* is the unified embedding dimension. Before entering the alignment module, the three types of features are first projected into a shared embedding space through linear projection layers to eliminate dimensional discrepancies across modalities.

During the cross-modal alignment process, visual features are used as the primary query, while concatenated environmental and trap features are used as the key and value to construct multi-head cross-attention. For the *h*-th attention head, the computation can be expressed as $Q_{h}=F_{uav}W_{h}^{Q}$, $K_{h}=\left[F_{env};F_{trap}\right]W_{h}^{K}$, and $V_{h}=\left[F_{env};F_{trap}\right]W_{h}^{V}$, where $W_{h}^{Q}$, $W_{h}^{K}$, and $W_{h}^{V}\in R^{d\times d_{h}}$ are learnable parameters, $d_{h}=d/H$, and *H* is the number of attention heads, which is typically set to 4 or 8. The attention weights are obtained through scaled dot-product computation, namely $\mathrm{Softmax}(Q_{h}K_{h}^{T}/\sqrt{d_{h}})$, thereby enabling adaptive selection of environmental and trap information conditioned on visual features. The outputs of all attention heads are concatenated and further transformed linearly to obtain the aligned feature $Z_{align}$. To enhance stability, residual connections and layer normalization are introduced into the structure, namely $Z_{align}=\mathrm{LayerNorm}\left(F_{uav}+\mathrm{MultiHeadAttn}\left(F_{uav},\left[F_{env};F_{trap}\right]\right)\right)$, thereby preventing gradient degradation during deep network training.

Furthermore, to strengthen explicit consistency constraints across modalities, a feature distribution matching mechanism is introduced into the alignment module, and semantic space convergence is achieved by minimizing the distance among different modality embeddings. We specifically define the alignment loss as $L_{align}={∥\mu \left(F_{uav}\right)-\mu \left(F_{env}\right)∥}_{2}^{2}+{∥\mu \left(F_{uav}\right)-\mu \left(F_{trap}\right)∥}_{2}^{2}$, where μ(·) denotes the feature mean mapping. The selection of this first-order mean distance over kernel-based or second-order metrics such as Maximum Mean Discrepancy (MMD) or CORAL is primarily motivated by the need for a computationally efficient regularization that establishes a stable semantic anchor for the attention mechanism. Given the extreme heterogeneity between spatial imagery and environmental time series, first-order alignment ensures global centroid convergence without over-constraining the complex, modality-specific feature distributions. This constraint effectively drives different modalities toward statistical consistency while preserving sufficient flexibility for nonlinear feature interaction, thereby improving the discriminative ability of the fused representation.

From a mathematical perspective, this module implements a conditional feature mapping, in which environmental and trap information are adaptively reconstructed under given visual observations, and its output can be regarded as an approximate modeling of the joint distribution $p(F_{env},F_{trap}|F_{uav})$. Compared with simple concatenation, this mechanism can adaptively adjust the contribution weights of different modalities to the final representation, thereby avoiding interference from redundant information while emphasizing key driving factors. For the task considered in this study, such a design offers significant advantages. On the one hand, visual features provide spatial distribution information, while environmental and trap features provide dynamic and local observational information; their collaborative modeling through attention mechanisms effectively compensates for the limitations of single-modality information. On the other hand, the multi-head structure captures cross-modal correlations from multiple subspaces, thereby enhancing the model’s capability to represent nonlinear relationships in complex agricultural scenarios. Therefore, the proposed cross-modal feature alignment module not only improves the effectiveness of feature fusion but also provides more consistent and discriminative input representations for subsequent density regression.

#### 3.3.3. Environment-Aware Enhancement Module

In the task considered in this study, the role of the environment-aware enhancement module is not simply to append variables such as temperature, humidity, and wind speed to visual features but rather to transform environmental information into dynamic control signals for visual representations through an explicit conditional modulation process, thereby enabling the model to adaptively reconstruct pest-response features according to current ecological conditions.

**Figure 3:**
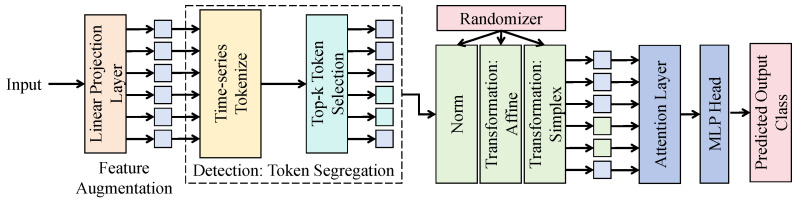
The environment-aware enhancement module dynamically injects environmental features into visual representations via temporal modeling and conditional affine modulation, enabling adaptive adjustment of spatial responses and channel importance.

As shown in Figure 3, this module receives two types of inputs. One is the feature map from the UAV visual encoder, denoted as $F_{v}\in R^{H\times W\times C}$, where *H* and *W* denote spatial height and width, respectively, and *C* denotes the number of channels. The other is the environmental sensor time series, denoted as $E\in R^{L\times D}$, where *L* denotes the temporal window length, and *D* denotes the dimensionality of environmental variables. First, the environmental sequence is transformed into an environmental embedding through linear projection and stacked one-dimensional temporal convolutions. The convolutional layers adopt a structure of channel expansion, temporal aggregation, and compression mapping. Specifically, the first layer maps the input channel from *D* to *C*, with kernels operating only along the temporal axis, and the output tensor remains of length *L* with channel dimension *C*. The second layer continues context aggregation along the temporal dimension, with the output width still being *L* and the channel dimension remaining *C*. The third layer obtains the environmental context vector $z\in R^{C}$ through global temporal aggregation. The computation can be expressed as

(10) $$U=\delta \left(\mathrm{BN}\left({\mathrm{Conv}}_{t}\left(\delta \left(\mathrm{BN}\left({\mathrm{Conv}}_{t}\left(\Pi (E)\right)\right)\right)\right)\right)\right),$$

(11) $$z=\frac{1}{L}\sum_{\ell }U_{\ell },$$

where Π(·) denotes linear projection, ${\mathrm{Conv}}_{t}$ denotes one-dimensional convolution along the temporal axis, BN(·) denotes normalization, and δ(·) denotes a nonlinear activation function. Subsequently, in order for environmental features to act upon the visual space, two parallel branches are constructed to generate a channel gating vector $a\in R^{C}$ and a bias vector $b\in R^{C}$, respectively. These are then expanded to the scale of H×W×C through a broadcasting mechanism and applied to visual features through affine enhancement, which can be written as

(12) $$a=\sigma \left(W_{a}z+b_{a}\right),\;b=W_{b}z+b_{b},$$

(13) $${\tilde{F}}_{v}=F_{v}⊙\left(1+B(a)\right)+B\left(b\right),$$

where σ(·) denotes the Sigmoid function, B(·) denotes broadcasting expansion from a channel vector to a spatial feature map, and ⊙ denotes element-wise multiplication. Furthermore, considering that the influence of environmental factors on different spatial positions is not completely uniform, a spatial response branch is introduced, in which ${\tilde{F}}_{v}$ is mapped through a 1×1 convolution to a single-channel weight map $M\in R^{H\times W}$, and the spatially enhanced result is generated jointly with the environmental vector:

(14) $$M=\sigma \left({\mathrm{Conv}}_{s}\left({\tilde{F}}_{v}\right)\right),$$

(15) $$F_{e}={\tilde{F}}_{v}⊙B\left(M\right),$$

where ${\mathrm{Conv}}_{s}$ denotes spatial convolution mapping. The advantage of this design lies in the fact that environmental information is first transformed into a stable context through temporal modeling and is then applied to visual representations through the dual mechanisms of channel recalibration and spatial reweighting, thereby simultaneously regulating which semantic channels are more important and which field regions should receive greater attention. From a mathematical perspective, this module can be regarded as a conditional affine transformation. When the gating vector *a* satisfies $a\in {\left(0,1\right)}^{C}$, the following inequality holds:

(16) $${∥{\tilde{F}}_{v}∥}_{F}\leq {∥F_{v}⊙\left(1+B(a)\right)∥}_{F}+{∥B(b)∥}_{F}\leq \left(1+{∥a∥}_{\infty }\right)∥F_{v}{∥}_{F}+\sqrt{HW}{∥b∥}_{2}.$$

Since ${∥a∥}_{\infty }<1$, the norm of the enhanced feature remains bounded and unstable amplification caused by environmental modulation is avoided, indicating good numerical stability of this module during training. Furthermore, if the loss function is differentiable with respect to the enhanced feature $F_{e}$, then according to the chain rule, the parameters of the environmental branch can receive supervision signals from the final density prediction through ∂L/∂a and ∂L/∂b, implying that this module is not an independent attachment but directly participates in end-to-end optimization. When applied to the pest density estimation task in this study, such a design explicitly injects ecological driving factors, such as temperature, humidity, wind speed, and rainfall, into visual feature learning so that the model no longer relies solely on static appearances such as leaf color changes or canopy texture but is able to make more reasonable density predictions in cases with similar visual symptoms but different environmental backgrounds, thereby effectively improving prediction accuracy and generalization in complex agricultural scenarios.

#### 3.3.4. Pest Density Regression Module

As can be observed from the module structure, the pest density regression module is not a simple terminal, fully connected prediction head but rather a structured prediction unit that simultaneously includes feature reorganization, saliency filtering, and global regression mapping. Its core idea is to perform decomposable modeling of fused features generated by preceding modules before final regression, thereby preserving dominant response components that are highly correlated with pest density while suppressing non-discriminative information related to background disturbances, local noise, and modality redundancy.

**Figure 4:**
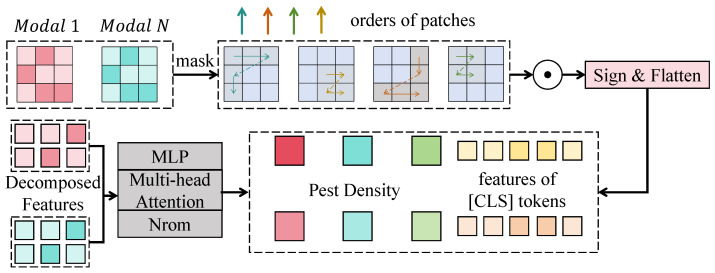
The pest density regression module transforms multimodal fused features into continuous pest density predictions through feature decomposition, global relation modeling, and multilayer perceptron mapping.

As shown in Figure 4, let the joint feature after cross-modal alignment and environment-aware enhancement be denoted as *F*, where *H* and *W* denote spatial height and width, and *C* denotes the number of channels. For subsequent global modeling, the feature map is first linearly rearranged and symbolically mapped into a sequence form *S*. Then, a learnable saliency mask generation branch is introduced, which produces a dominant response weight map *M* through position-wise mapping and decomposes the features into density-sensitive and complementary components:

(17) $$S^{+}=M⊙S,$$

(18) $$S^{-}=\left(1-M\right)⊙S,$$

where *M* is generated by a lightweight mapping network. The design of this decomposition mechanism is driven by the theoretical requirement of optimal feature selection and the principles of the Information Bottleneck (IB). From a feature selection perspective, the mask *M* functions as an adaptive soft-gate that identifies and extracts features possessing high mutual information with the target density *y*, such as localized damage textures and canopy sparsity patterns. Furthermore, this process can be interpreted through the lens of the Information Bottleneck principle, where the decomposition acts as a controllable bottleneck that minimizes the inclusion of task-irrelevant entropy—such as soil background, shadow variations, and sensor-induced redundancy—while maximizing the retention of sufficient statistics for the regression task. By segregating the representation into $S^{+}$ and $S^{-}$, we explicitly enhance the signal-to-noise ratio (SNR) of the input to the regression head. This ensures that the global relation aggregation layer can focus on a refined manifold of density-sensitive signals, effectively preventing the regression accuracy from being compromised by high-entropy environmental noise commonly found in complex field scenarios.

In terms of network structure, the regression module consists of three parts. The first part is the feature decomposition layer, which redistributes semantic information while maintaining spatial dimensions. The second part is the global relation aggregation layer, in which salient features $S^{+}$ and complementary features $S^{-}$ are linearly mapped and fed into a multi-head attention unit to establish global dependencies. Let the mapped query, key, and value be denoted as $Q_{r}$, $K_{r}$, and $V_{r}$, respectively; then the aggregation process is

(19) $$R=\mathrm{Softmax}\left(\frac{Q_{r}K_{r}^{⊤}}{\sqrt{d}}\right)V_{r},$$

where *d* denotes the hidden-space dimension. The aggregated result is then passed together with a global token vector through normalization and a feed-forward mapping layer to form the final global regression representation $g\in R^{C}$. The third part is a multilayer perceptron regression head, which adopts a three-layer progressively compressed structure. Its input dimension is *C*, and the hidden-layer dimensions are successively compressed into lower-dimensional semantic subspaces. Normalization, nonlinear activation, and dropout are configured between layers to improve nonlinear fitting ability and generalization performance. The final output is the scalar pest density prediction $\hat{y}$, and its mapping process can be expressed as

(20) $$u=\varphi \left(W_{a}g+b_{a}\right),$$

(21) $$v=\varphi \left(W_{b}u+b_{b}\right),$$

(22) $$\hat{y}=W_{c}v+b_{c},$$

where φ(·) denotes the nonlinear activation function. To ensure stable fitting of the regression module for the continuous variable of pest density, a robust regression objective is adopted during training, in which the deviation between the predicted value and the ground-truth density *y* is constrained in a smooth form:

(23) $$L_{d}=\left\{\begin{array}{cc} \frac{1}{2}{\left(\hat{y}-y\right)}^{2}, & \left|\hat{y}-y\right|<\epsilon \\ \epsilon \left|\hat{y}-y\right|-\frac{1}{2}\epsilon^{2}, & \mathrm{otherwise} \end{array}\right..$$

This loss maintains second-order differentiability when the error is small and becomes a linear penalty when the error is large, thereby ensuring local convergence accuracy while preventing excessively large gradients caused by outlier samples. Furthermore, from a mathematical perspective, the regression mapping can be shown to possess local stability with respect to input perturbations. If the weights of all linear layers are bounded and the activation function satisfies Lipschitz continuity, then there exists a constant κ such that for any two input representations *g* and $\tilde{g}$, the following inequality holds:

(24) $$\left|f\left(g\right)-f\left(\tilde{g}\right)\right|\leq \kappa {∥g-\tilde{g}∥}_{2},$$

where f(·) denotes the entire regression head. This indicates that when fused features undergo small perturbations, the prediction output will not exhibit unstable jumps, thereby ensuring continuous prediction capability under complex agricultural scenarios. Applied to the pest density estimation task considered in this study, the advantage of this module lies in its ability to first strengthen dominant features related to density through saliency decomposition, then capture spatial relationships among different damaged regions through global relation aggregation, and finally achieve accurate projection from high-dimensional joint representations to continuous pest density through multilayer nonlinear mapping. Therefore, compared with directly using a single-layer regressor or simple average pooling, this module is more suitable for handling real-world problems characterized by uneven field-scale pest distributions, strong local anomalous responses, and complex coupling of multimodal information.

## 4. Results and Discussion

### 4.1. Experimental Setup

The experiments were performed on a high-performance computing node featuring an Intel Xeon Platinum 8358P CPU, 128 GB of DDR4 RAM (Intel, Santa Clara, CA, USA), and an NVIDIA GeForce RTX 3090 GPU (24 GB VRAM) (Nvidia, Santa Clara, CA, USA). High-speed NVMe SSDs were used to optimize data I/O for the multimodal datasets.

The software environment was built on Ubuntu 20.04 using Python 3.9. The PDEF framework was implemented using PyTorch 2.1.0 with CUDA 12.1 acceleration. Other critical libraries included NumPy 1.24.3 and Pandas 2.0.2 for data processing, OpenCV 4.8.0 and Albumentations 1.3.1 for image augmentation, and Scikit-learn 1.2.2 for metric evaluation. Training progress and model convergence were monitored via TensorBoard 2.13.0.

The dataset was partitioned into training, validation, and testing sets with a 70:15:15 ratio. The model was trained for 100 epochs using the Adam optimizer with a batch size of 16. The initial learning rate was $1\times 10^{-4}$, adjusted by a cosine annealing scheduler. We employed Mean Squared Error (MSE) as the loss function and applied an early stopping strategy to mitigate overfitting. To ensure evaluation stability, 5-fold cross-validation was utilized, and final results were averaged across multiple runs.

### 4.2. Baseline and Evaluation Metrics

To comprehensively evaluate the effectiveness of the proposed method, several representative baseline models were selected for comparative experiments. The linear regression model [50] is characterized by its simple structure and high computational efficiency, enabling rapid establishment of a fundamental mapping between input and output and providing a basic reference for subsequent models. The random forest model [51], based on ensemble learning of multiple decision trees, exhibits strong nonlinear modeling capability and robustness and performs stably under complex data conditions. The multilayer perceptron model [52] utilizes multiple nonlinear transformations to enhance feature representation, demonstrating effective fitting ability for complex relationships. The convolutional neural network based on ResNet50 [53] is capable of extracting rich spatial structures and texture features from UAV imagery, achieving strong performance in visual modeling tasks. The LSTM model [54] employs a gating mechanism to model temporal sequences, effectively capturing the dynamic variations of environmental variables. The CNN–LSTM fusion model [55] integrates the advantages of spatial and temporal feature modeling, enabling preliminary fusion of multi-source information. The Transformer-based fusion model [56] leverages a self-attention mechanism to model global dependencies, allowing efficient capture of complex relationships across different modalities and thereby improving overall predictive performance.

For the pest density estimation task, mean absolute error (MAE) was employed to measure the average deviation between predicted values and ground truth; root mean squared error (RMSE) was used to quantify the overall magnitude of errors with higher sensitivity to large deviations; and the coefficient of determination $R^{2}$ was adopted to evaluate the model’s ability to explain variance and capture data trends. The mathematical definitions of these evaluation metrics are given as follows. Let the ground-truth pest density be ${\left\{y_{i}\right\}}_{i=1}^{N}$ and the predicted values be ${\left\{{\hat{y}}_{i}\right\}}_{i=1}^{N}$:

(25) $$\mathrm{MAE}=\frac{1}{N}\sum_{i=1}^{N}\left|y_{i}-{\hat{y}}_{i}\right|,$$

(26) $$\mathrm{RMSE}=\sqrt{\frac{1}{N}\sum_{i=1}^{N}{\left(y_{i}-{\hat{y}}_{i}\right)}^{2}},$$

(27) $$R^{2}=1-\frac{\sum_{i=1}^{N}{\left(y_{i}-{\hat{y}}_{i}\right)}^{2}}{\sum_{i=1}^{N}{\left(y_{i}-\bar{y}\right)}^{2}},$$

where *N* denotes the number of samples, $y_{i}$ represents the ground-truth pest density of the *i*-th sample, ${\hat{y}}_{i}$ denotes the corresponding predicted value, and $\bar{y}$ is the mean of all ground-truth values, given by $\bar{y}=\frac{1}{N}\sum_{i=1}^{N}y_{i}$. The absolute value operator $\left|\cdot \right|$ is used in MAE to measure deviation, while the squared term ${\left(y_{i}-{\hat{y}}_{i}\right)}^{2}$ emphasizes larger errors in RMSE.

### 4.3. Comparison with Baseline Methods

The purpose of this experiment is to systematically evaluate the effectiveness of the proposed method for pest density estimation and to verify the necessity of multimodal fusion and structured modeling from different modeling paradigms by comparing with several representative baseline models. Specifically, the experiment covers traditional statistical methods, classical machine learning models, single-modality deep learning approaches, and multimodal fusion models, thereby establishing a comprehensive performance comparison framework.

**Figure 5:**
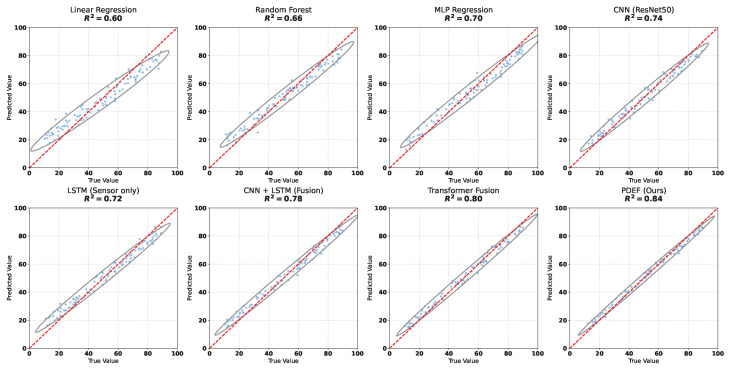
Scatter plots comparing true and predicted pest density values across different models.

As shown in Table 2 and Figure 5, the linear regression model exhibits the weakest performance across all metrics, indicating its limited capability in modeling complex nonlinear relationships and its reliance on simple linear trends. The random forest model improves performance through nonlinear tree-based structures, yet the lack of explicit modeling for spatial structure and temporal dependency constrains its effectiveness. The MLP model further reduces prediction error through multilayer nonlinear transformations; however, its insensitivity to input structure limits the utilization of spatial information in imagery. The ResNet50-based CNN effectively extracts spatial features from crop damage patterns, resulting in improved performance compared with traditional methods. The LSTM model captures the temporal dynamics of environmental variables but remains inferior to visual models due to the absence of spatial information. The CNN+LSTM fusion model integrates spatial and temporal features, leading to notable performance gains. The Transformer-based fusion model further enhances performance by modeling global dependencies across modalities. While the pre-trained CLIP features provide robust visual–semantic representations and achieve competitive results, the proposed PDEF achieves the best performance across all metrics. This demonstrates that PDEF’s domain-specific design—specifically its ability to model the complex coupling between localized IoT environmental sequences and UAV-based spatial damage patterns—offers superior capability in multimodal feature integration for agricultural scenarios compared to general-purpose multimodal models.

From a theoretical perspective, performance differences among models originate from their distinct capacities in modeling data distributions and feature relationships. Linear regression assumes a linear mapping between inputs and outputs, which restricts its applicability in complex agricultural scenarios characterized by nonlinear interactions. Random forest approximates nonlinear functions through piecewise partitioning, but its decision boundaries are inherently discrete and lack global continuity, leading to suboptimal performance in continuous density estimation tasks. The MLP possesses universal approximation capability but lacks structural priors, resulting in inefficient utilization of spatial and temporal information. The convolutional structure in ResNet introduces local receptive fields and weight sharing, enabling effective modeling of spatial patterns in UAV imagery. LSTM models temporal dependencies through gated state updates, capturing environmental dynamics, yet its single-modality input constrains its representation power. The CNN–LSTM fusion model combines complementary features but remains limited to shallow fusion. The Transformer-based model employs attention mechanisms to establish global interactions across modalities, thereby enhancing representation capability. Building upon this, PDEF introduces cross-modal alignment and environment-aware enhancement mechanisms, enabling both representation-level alignment and semantic-level modulation across modalities. Such improvements in feature space structure and information interaction allow the model to more accurately approximate the complex functional relationship underlying pest density, leading to superior performance across all evaluation metrics.

### 4.4. Ablation Study

The purpose of the ablation study is to systematically evaluate the contribution of each key component in the PDEF framework, demonstrating that performance improvement arises from the collaborative effect of multiple modules rather than a single structural element. By progressively removing the cross-modal alignment module, the environment-aware enhancement module, the feature decomposition mechanism, and the multimodal fusion structure, the impact of each component on prediction performance can be clearly observed.

**Figure 6:**
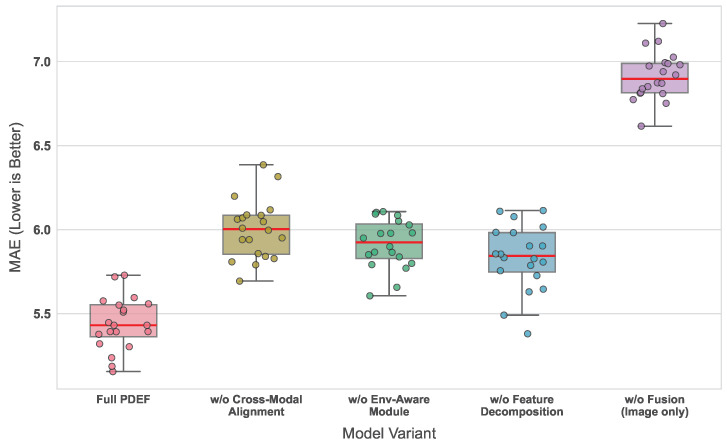
Box plots of MAE distributions under different ablation settings.

As shown in Table 3 and Figure 6, the full model achieves the best performance across all evaluation metrics, while the removal of any module results in performance degradation to varying degrees. The most significant performance drop is observed when only image data are used, highlighting the fundamental importance of multimodal information for modeling complex pest distributions. Removing the cross-modal alignment module leads to a clear performance decline, indicating that simple feature concatenation is insufficient for effective fusion of heterogeneous data. The removal of the environment-aware module and the feature decomposition mechanism also results in consistent performance degradation, although the magnitude is relatively smaller, suggesting that these components play an enhancing role in refined modeling stages. From a theoretical perspective, the differences in module contributions stem from their distinct roles in structuring the feature space and facilitating information interaction. The cross-modal alignment module enforces a unified semantic space, reducing distribution discrepancies across modalities and enabling effective integration of heterogeneous information. The environment-aware module introduces conditional modulation, allowing environmental variables to dynamically influence feature representation, thereby enhancing responsiveness to temporal variations. The feature decomposition mechanism separates relevant signals from noise, improving the focus of the regression stage on informative features. When multimodal fusion is entirely removed, the model degenerates into a single-modality mapping, which can only approximate spatial patterns while ignoring temporal and environmental dependencies, resulting in significantly reduced fitting capability. Overall, these modules collectively optimize feature representation, alignment, and function approximation, enabling more accurate modeling of the complex nonlinear relationship underlying pest density.

### 4.5. Sensitivity Analysis of Environmental Factors

To further enhance the interpretability of the PDEF framework and understand the specific contributions of different environmental variables, we conducted a sensitivity analysis on the core factors within the IoT sequences. The purpose of this experiment is to quantify how individual variables—namely temperature, humidity, wind speed, and precipitation—influence the final pest density estimation. By systematically perturbing each input feature while keeping others constant, we calculated the relative increase in prediction error (MAE and RMSE) and derived a normalized importance score for each factor. This analysis provides a transparent view of the model’s internal decision-making logic, ensuring that the environment-aware modulation aligns with established ecological and agronomic principles.

The experimental results presented in Table 4 reveal that temperature and humidity are the most influential drivers in the pest density estimation process, accounting for over 65% of the total environmental importance. This finding is highly consistent with biological research indicating that hydrothermal conditions are primary determinants of pest reproductive cycles and population dynamics. Wind speed and precipitation exhibit lower but statistically significant impacts, primarily by influencing UAV flight stability and local pest migration patterns. By demonstrating that the PDEF framework prioritizes environmental factors in a manner that reflects real-world ecological dependencies, we provide agricultural practitioners with a more reliable and interpretable tool. This sensitivity analysis effectively bridges the gap between black-box deep learning and practical agronomic knowledge, thereby building greater trust in the model’s localized predictions.

### 4.6. Performance Comparison Under Different Input Modalities

The purpose of this experiment is to analyze the contribution of different modalities to pest density estimation from a data perspective and to further validate the necessity and effectiveness of multimodal fusion. By evaluating single-modality inputs, pairwise combinations, and full multimodal fusion, the influence of different information sources on model performance can be systematically assessed.

**Figure 7:**
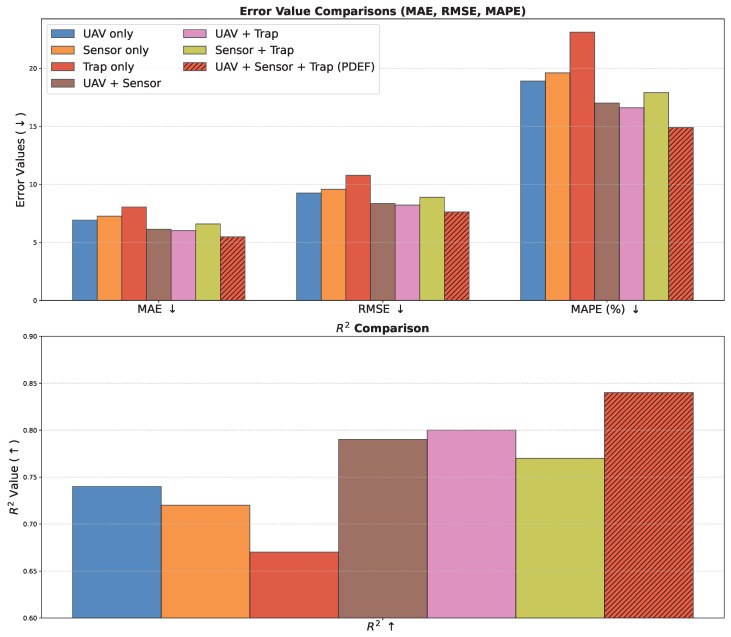
Performance comparison of different input modality combinations in terms of MAE, RMSE, MAPE, and $R^{2}$.

As shown in Table 5 and Figure 7, single-modality models exhibit relatively weaker performance, among which UAV imagery achieves better results than sensor and trap data, indicating the strong capability of spatial visual information in representing pest distribution. Sensor-only models capture environmental trends but lack spatial information, resulting in slightly inferior performance compared with visual models. Trap-only models perform the worst due to their sparse and localized observations. When dual-modality fusion is applied, performance improves significantly across all metrics, with the UAV and trap combination achieving the best results among pairwise inputs, demonstrating strong complementarity between spatial distribution and local observations. The UAV and sensor combination also performs well, while the sensor and trap combination shows relatively limited improvement due to the absence of direct spatial information. The full multimodal PDEF model achieves the best performance, validating the effectiveness of integrating multi-source information. From a theoretical perspective, the differences across modalities arise from their ability to approximate different aspects of the target function. UAV imagery provides continuous spatial observations, enabling effective modeling of local dependencies through convolutional structures. Sensor data capture temporal dynamics, representing indirect modeling of pest evolution processes but lacking spatial localization. Trap data are sparse, discrete observations with strong statistical significance but limited spatial coverage. In dual-modality fusion, complementary information improves approximation capability in a higher-dimensional feature space, although the absence of unified alignment may still introduce redundancy. In contrast, multimodal fusion integrates spatial, temporal, and local observational information, enabling a more comprehensive representation of pest density generation mechanisms. This corresponds to a multivariate joint modeling process, significantly improving approximation accuracy and generalization. Therefore, the results demonstrate the necessity and effectiveness of multimodal collaborative modeling in complex agricultural environments.

### 4.7. Discussion

#### 4.7.1. Theoretical Analysis of Model Properties and Convergence

To further substantiate the scientific foundation of the PDEF framework, it is essential to analyze its theoretical properties beyond empirical performance. The proposed architecture exhibits several key mathematical characteristics that ensure its effectiveness in complex agricultural sensing tasks. First, the convergence of the model is theoretically supported by the design of the loss function and the structural constraints of the regression head. By employing the smooth L1 loss for pest density estimation, we ensure that the objective function is both continuous and differentiable, with gradients that are bounded for large errors and decrease linearly as the error approaches zero. This prevents gradient explosion during the initial stages of training on high-density outlier samples and promotes local convergence stability. Furthermore, if we consider the entire PDEF as a functional mapping f, its convergence is facilitated by the Lipschitz continuity of the constituent modules. Given that the convolutional layers and the multilayer perceptron employ bounded activation functions and normalized weight matrices, the overall mapping satisfies $|f\left(g\right)-f\left(\tilde{g}\right)|\leq \kappa ∥g-\tilde{g}∥$, where κ is the Lipschitz constant. This property ensures that the model output remains stable under small perturbations in the multimodal input space.

The behavior of the model under different types of input data is governed by the synergistic interaction between the cross-modal alignment and the environment-aware enhancement modules. From the perspective of information theory, the saliency mask generation in the regression module acts as an adaptive information bottleneck. It selectively preserves features with high mutual information regarding the pest density while discarding task-irrelevant entropy from the background imagery or sensor noise. When encountering high-variance environmental data—such as rapid fluctuations in humidity or illumination—the environment-aware enhancement module functions as a dynamic regularizer. It modulates the visual feature space using environmental signals as prior conditions, effectively shifting the internal representation to maintain consistency across varying ecological states. This theoretical mechanism explains the framework’s superior robustness compared to conventional concatenation-based fusion methods. Additionally, the cross-modal alignment loss $L_{align}$ forces the heterogeneous embeddings from UAV, IoT, and trap sources into a shared Hilbert space, ensuring that the joint representation is not dominated by a single modality. This balanced information flow is critical for maintaining model performance in scenarios where one data source may be temporarily degraded or missing, thereby providing a theoretically grounded pathway for reliable environmental state estimation in intelligent agricultural systems.

#### 4.7.2. Model Generalization Capability

The adaptability of the PDEF framework to diverse climate zones and crop types is fundamentally supported by its hierarchical modular architecture. To facilitate the application of PDEF to new agricultural scenarios, we propose a multi-stage transfer learning strategy. First, the convolutional backbone, which is pre-trained on large-scale agricultural datasets, can be partially frozen to retain low-level spatial features while fine-tuning the higher-level alignment and regression layers with small-scale localized data. This parameter-efficient fine-tuning approach significantly reduces the data requirements for new regions. Second, the environment-aware enhancement module serves as a dynamic domain adaptation layer. By utilizing localized ecological signals to generate affine transformation parameters, the framework can calibrate visual features in real-time to account for variations in pest phenology and crop reflectance across different geographic regions. This design effectively addresses the covariate shift problem common in cross-regional agricultural sensing. From the perspective of the Information Bottleneck theory, the feature decomposition layer provides an additional layer of robustness by segregating density-sensitive signals S+ from the complementary background noise S−. This ensures that only the most invariant and task-relevant features are propagated to the final regression head, suggesting a strong potential for few-shot transfer when applied to new pest species with similar damage morphologies.

#### 4.7.3. Practical Deployment in Intelligent Agricultural Systems

The proposed method demonstrates clear practical value in real-world agricultural production scenarios, particularly for large-scale crop fields and orchards requiring continuous pest monitoring. In traditional practice, pest assessment is typically conducted through manual field inspection or simple trap-based counting, which is labor-intensive and fails to capture spatial variability across large areas. For instance, in horticultural production regions of Bayannur, Inner Mongolia, pest infestation often exhibits spatial clustering, with significant variation across different plots. Decisions based solely on a limited number of trap observations may lead to imprecise interventions, resulting in either excessive pesticide application or delayed control measures. By integrating UAV imagery, trap data, and environmental information, the proposed multimodal framework enables the generation of more continuous and fine-grained pest distribution maps at the field scale, providing more reliable decision support for agricultural practitioners.

To evaluate the feasibility of real-world application, we assessed the computational costs and architectural scale of the PDEF framework. The total number of learnable parameters in the model is approximately 28.4 million. For training, a workstation equipped with an NVIDIA RTX 3090 GPU requires approximately 6.5 h on the full multimodal dataset. For practical deployment, the inference efficiency is a critical factor; our framework achieves an average inference latency of 48 ms per data sample on the same hardware. The peak memory footprint during inference is recorded at 4.2 GB. While these metrics demonstrate suitability for edge computing platforms, we further acknowledge that deployment on resource-constrained UAVs can be optimized through lightweighting strategies such as knowledge distillation and structural pruning. By distilling knowledge from the full PDEF into a smaller student network or pruning non-essential attention heads, the model size and power consumption can be significantly reduced without substantial loss in estimation accuracy, thereby ensuring real-time performance in field-based intelligent agricultural systems.

In practical deployment, the method can be integrated into intelligent agricultural systems to form a closed-loop workflow, including aerial monitoring, data fusion, density estimation, and precision control. For example, in orchard management, UAVs can periodically capture canopy imagery, while the system analyzes leaf color variations and damage patterns, combining these observations with trap counts and environmental measurements to estimate pest density dynamically. When a specific area reaches a predefined threshold, targeted interventions can be recommended, avoiding uniform pesticide application across the entire field. This approach reduces chemical usage and environmental impact while improving control efficiency. In greenhouse environments, where pest propagation is more sensitive to environmental fluctuations, the environment-aware mechanism enables more accurate modeling of pest growth under varying temperature and humidity conditions. Such a multimodal monitoring strategy enhances agricultural intelligence and contributes to sustainable and environmentally friendly pest management.

#### 4.7.4. Broader Implications for Agricultural Systems

The transition from experience-driven to data-driven agricultural management represents a fundamental shift in productivity and resource allocation. By providing accurate field-scale pest density quantification, the proposed framework enables stakeholders to move beyond uniform, prophylactic pesticide application toward targeted, threshold-based interventions. This precision optimizes input-output efficiency by reducing chemical expenditures and labor costs while maintaining yield stability. Beyond the production level, the high-resolution sensing data enhances the transparency and resilience of agricultural supply chains. Real-time monitoring of biological risks allows upstream and downstream participants to proactively adapt to yield fluctuations, facilitating better logistics scheduling and market supply-demand balance. The digitalization of these field signals provides a critical technological foundation for building adaptive agri-food systems capable of responding to environmental and market uncertainties.

### 4.8. Limitations and Future Work

Despite its performance, several practical challenges must be addressed for large-scale deployment in complex agricultural environments. Illumination variations in UAV imagery, often caused by shifting cloud cover or sun angles, can introduce radiometric noise and affect the consistency of canopy feature extraction. To mitigate this, we plan to incorporate advanced color normalization techniques and light-invariant data augmentation during the training phase to improve model robustness. Another critical issue is sensor data loss due to transmission failures or battery depletion in IoT nodes. While we currently utilize temporal interpolation and neighborhood-based imputation to handle missing environmental values, the integration of generative adversarial networks for data recovery remains a promising future direction. Furthermore, the effectiveness of the PDEF framework is sensitive to trap placement strategies. In our study, traps were arranged in a grid-like pattern, but this may not be optimal for irregularly shaped fields or areas with localized microclimates. We intend to investigate the use of spatial sampling theory and Voronoi tessellation to optimize trap density and placement, ensuring maximum representation of pest population dynamics. To specifically address the model sensitivity in edge cases, we analyzed performance across density-based subsets of the test set. The results indicate a measurable degradation in accuracy during extremely low-density infestations and sudden outbreaks, where visual cues are subtle and environmental drivers shift rapidly. To enhance early warning capabilities, future work will focus on incorporating temporal forecasting to model outbreak probabilities and utilizing few-shot learning techniques to improve the framework’s adaptability to rare or sudden biological events. Additionally, the regional focus of the current study may limit its immediate generalization across highly diverse climate zones. Future research will address these challenges by developing robust domain-adversarial training techniques to improve model invariance.

## 5. Conclusions

In the context of precision agriculture, accurate field-scale pest monitoring remains a challenging task. To address the difficulty of achieving quantitative pest assessment at the farmland scale, a multimodal pest density estimation framework, namely PDEF, is proposed by integrating UAV-based remote sensing, trap observations, and environmental sensor data. Starting from plant phenotypic variations and ecological driving mechanisms, intelligent modeling and prediction of pest occurrence processes are achieved. By constructing a multi-source agricultural monitoring dataset and designing cross-modal feature alignment, environment-aware enhancement, and structured regression modules, inconsistencies among different data modalities in spatial, temporal, and semantic dimensions are effectively mitigated. As a result, collaborative learning in a unified representation space is enabled, improving adaptability to complex agricultural scenarios. Experimental results demonstrate that the proposed method significantly outperforms several mainstream models in pest density estimation, achieving notable reductions in MAE, RMSE, and MAPE, while attaining $R^{2}=0.84$, indicating superior fitting capability and stability. In both multimodal fusion and ablation experiments, each module is validated to be effective, reflecting the overall advantages of the model in terms of accuracy and robustness. From the perspective of integrating plant phenotypic analysis and environmental perception, this study provides a novel artificial intelligence-driven approach for pest monitoring and crop management.

## Figures and Tables

**Table 1:** Summary of multimodal agricultural pest monitoring dataset.

Data Modality	Data Source	Sampling Frequency	Data Volume
UAV imagery	UAV platform with RGB camera	Every 5–7 days	18,500 images
Trap counts	Pheromone traps deployed in fields	Every 3–5 days	12,000 records
Environmental sensors	Field IoT weather stations	Every 10–30 min	2.6 × 10^6^ records
Temporal coverage	Entire growing season	–	April 2023–October 2024
Spatial coverage	Multiple agricultural fields	–	15+ field plots

**Table 2:** Comparison with baseline methods on pest density estimation. Down arrows indicate that a lower value is better. Up arrows indicate that a higher value is better.

Method	MAE ↓	RMSE ↓	MAPE (%) ↓	*R*^2^ ↑
Linear Regression	8.91	11.38	24.7	0.60
Random Forest	8.12	10.65	22.3	0.66
MLP Regression	7.58	10.02	20.8	0.70
CNN (ResNet50)	6.92	9.24	18.9	0.74
LSTM (Sensor only)	7.25	9.57	19.6	0.72
CNN + LSTM (Fusion)	6.34	8.51	17.2	0.78
Transformer Fusion	6.01	8.16	16.5	0.80
CLIP	5.82	7.95	15.8	0.81
**PDEF (Ours)**	**5.47**	**7.62**	**14.9**	**0.84**

**Table 3:** Ablationstudy of different modules in PDEF. Down arrows indicate that a lower value is better. Up arrows indicate that a higher value is better.

Model Variant	MAE ↓	RMSE ↓	MAPE (%) ↓	*R*^2^ ↑
Full PDEF	**5.47**	**7.62**	**14.9**	**0.84**
w/o Cross-Modal Alignment	6.05	8.28	16.8	0.80
w/o Environment-Aware Module	5.92	8.14	16.3	0.81
w/o Feature Decomposition	5.84	8.05	15.9	0.82
w/o Multimodal Fusion (Image only)	6.92	9.24	18.9	0.74

**Table 4:** Sensitivity analysis and feature importance of individual environmental factors. Down arrows indicate that a lower value is better. Up arrows indicate that a higher value is better.

Environmental Factor	MAE Increase ↑	RMSE Increase ↑	Importance Score
Temperature	1.12	1.45	0.38
Humidity	0.89	1.18	0.29
Wind Speed	0.45	0.62	0.18
Precipitation	0.32	0.41	0.15

**Table 5:** Performance comparison under different input modalities. Down arrows indicate that a lower value is better. Up arrows indicate that a higher value is better.

Input Modality	MAE ↓	RMSE ↓	MAPE (%) ↓	*R*^2^ ↑
UAV only	6.92	9.24	18.9	0.74
Sensor only	7.25	9.57	19.6	0.72
Trap only	8.05	10.78	23.1	0.67
UAV + Sensor	6.12	8.34	17.0	0.79
UAV + Trap	6.01	8.21	16.6	0.80
Sensor + Trap	6.58	8.88	17.9	0.77
**UAV + Sensor + Trap (PDEF)**	**5.47**	**7.62**	**14.9**	**0.84**

## Data Availability

The data presented in this study are available on request from the corresponding author. To facilitate reproducibility and support future research by the scientific community, the complete source code for the PDEF framework is publicly available at https://github.com/Aurelius-04/PDEF.git (accessed on 1 May 2026).
